# Toxicity Assessment of Herbal Medicine Using Zebrafish Embryos: A Systematic Review

**DOI:** 10.1155/2019/7272808

**Published:** 2019-11-06

**Authors:** Chanika D. Jayasinghe, Uthpala A. Jayawardena

**Affiliations:** Department of Zoology, Faculty of Natural Sciences, The Open University of Sri Lanka, Nawala, Nugegoda, Sri Lanka

## Abstract

Herbal remedies have been practiced by humans over centuries and therefore possess time-proven safety. However, it is imperative to evaluate the toxic effects of herbal medicine to confirm their safety, particularly when developing therapeutic leads. Use of laboratory animals such as rats, mice, and rabbits was considered as gold standard in herbal toxicity assessments. However, in the last few decades, the ethical consideration of using higher vertebrates for toxicity testing has become more contentious. Thus, possible alternative models entailing lower vertebrates such as zebrafish were introduced. The zebrafish embryotoxicity model is at the forefront of toxicology assessment due to the transparent nature of embryos, low cost, short cycle, higher fecundity, and genetic redundancy to the humans. Recently, its application has been extended to herbal toxicology. The present review intends to provide a comprehensive assembly of studies that applied the zebrafish embryo model for the assessment of herbal toxicity. A systematic literature survey was carried out in popular scientific databases. The literature search identified a total of 1014 articles in PubMed = 12, Scopus SciVerse® = 623, and Google Scholar = 1000. After screening, 25 articles were included in this review, and they were categorized into three groups in which the zebrafish embryotoxicity assay has been applied to investigate the toxicity of (1) polyherbal formulae/medical prescription (2 full texts), (2) crude extracts (12 full texts), and (3) phytocompounds/isolated constituents (11 full texts). These studies have investigated the toxicity of 6 polyherbal formulae, 16 crude extracts, and more than 30 phytocompounds/isolated constituents using the zebrafish embryotoxicity model. Moreover, this model has explicated the teratogenic effects and specific organ toxicities such as the kidney, heart, and liver. Furthermore, in some studies, the molecular mechanisms underlying the toxicity of herbal medicine have been elucidated. This comprehensive collection of scientific data solidifies the zebrafish embryo model as an effective model system for studying toxicological effects of a broad spectrum of herbal remedies. Henceforth, it provides a novel insight into the toxicity assessment of herbal medicine.

## 1. Introduction

Herbal medicine, with cultural and traditional roots extended over the history of many countries of the developing world is still pertinent and customary. An estimated 80% of the population of some Asian and African countries at present use herbal medicines as the mainstay for their primary health care needs [1]. In Africa, up to 90% and in India, 70% of the population depends on traditional medicine [1]. Recently, herbal medicine has gained resurgent interest in the Western world due to the evidence of better patient tolerance and holistic treatment approach.

Unlike synthetic drugs, herbal remedies have been consumed by humans over centuries and possess time-proven safety. However, toxicological assessment is paramount in herbal medicine to identify adverse effects to safeguard human beings [2]. Evaluation of toxicity at preclinical and clinical stages of drug discovery will facilitate the identification of toxicants which can be discarded or modified into a safer alternative [3].

Both crude and acute constituents of herbal preparation/medicine are screened for toxicity [4]. Toxicity screening is conducted with animal and nonanimal models prior to clinical application. Animal models are considered as gold standards in toxicology testing since the whole animal is typically closely correlated to human toxicity and more importantly incorporates pharmacokinetics, absorption, distribution, and metabolism [4]. Test organisms range from invertebrates such as brine shrimp to mammals such as mice, rats, guinea pigs, and rabbits [4]. Different exposure routes including oral gavage, inhalation/mucosal, and dermal or by injection into the bloodstream, abdomen, or the muscles could be evaluated following the administration of a test product [4].

Although reliable data for extrapolating toxicant effects to humans are obtained through laboratory rodent studies, these are expensive, time consuming, and more restricted by animal rights and ethics concerns [5]. The pain, distress, and death experienced by the animals during scientific experiments have been a debate for a long time. The determination of LD_50_ involves a large number of animals suffering higher mortality [6]. Ethical consideration of animals involved in research gave rise to the adaptation of 3R's principal introduced by Russell et al. [7]. This urged to reduce the number of animals, refine the test methods used in order to minimize pain and suffering of experimental animals, and replace animal tests with validated alternatives where possible [8]. In addition, both lower vertebrate and invertebrate organisms are widely used as an alternatives for higher vertebrates for toxicity testing [5]. Organisms such as zebrafish, brine shrimp, and daphnia are the most commonly used alternative organisms for herbal drug toxicological evaluation [5]. Among them, the zebrafish embryonic toxicity model has gained considerable attention as an alternative screening platform to evaluate the toxicity of bioactivity compounds [9].

*Danio rerio*, commonly called as the zebrafish, is a small freshwater fish with an approximate length of 2–4 cm. Small size, short life cycle, and high fecundity favour its laboratory uses as a test organism [9]. Zebrafish has been extensively used for genetic development, transgenesis, and toxicological studies [10]. Zebrafish has become a popular model for genetic research due to the high conservation of gene function between zebrafish and humans. It has been reported 87% of genetic similarity exists between the zebrafish and human [11].

Both adults and embryos of the zebrafish are used as laboratory models [11]. However, the embryo stages are preferably used for toxicological assessments particularly due to the transparent nature of the egg which allows the direct observation of developmental stages and assessment of endpoint in toxicity. In addition, small embryos with major organ primordia developed within 24 hours and the possibility of *in vitro* fertilization and development allowing easy observation and manipulation are other advantages. Furthermore, low cost, short cycle, higher fecundity, the requirement of small quantities of test compounds, and high throughput screening also make it a highly suitable and successful model to be used in toxicity studies [12, 13].

The zebrafish embryotoxicity model is at the forefront of the ecotoxicological assessments [14]. The OECD (Organization for Economic Co-operation and Development) has developed guidelines to evaluate the embryotoxicity effects of certain compounds on the early 96 hr of developmental stages (acute toxicity testing) ((OECD) test no. 236, 2013) [15].

Recently, the zebrafish embryotoxicity model has received considerable attention in toxicity assessment of natural products and herbal medicine. Use of the zebrafish embryotoxicity model reduces the usage of higher vertebrates into toxicity studies. The zebrafish embryotoxicity model is more suitable for isolated compounds from herbal medicine as the test can be performed with small quantities. The transparent nature of embryos allows evaluating the effects of compounds on various organs, including the heart, brain, intestine, pancreas, cartilage, liver, and kidney without complicated processing [16]. Furthermore, this model is more suitable for detecting the off-target effects or multiple targets due to the holistic action of natural products [17]. However, the use of the zebrafish embryotoxicity model for herbal toxicity field requires more attention.

There is a pertinent need to renew scientific enthusiasm toward incorporating the zebrafish embryotoxicity model in toxicological evaluation on herbal medicine. Hence, this review intends to provide a comprehensive account of available scientific evidence of the application of the zebrafish embryos to assess the herbal toxicity. The scientific evidence gathered herein will be encouraged to apply the zebrafish embryotoxicity for herbal drug toxicity analysis. Furthermore, this model will allow understanding the molecular mechanism underlying the toxicity of the natural products. It is anticipated that this work will highlight a novel vista in herbal toxicology that uses zebrafish embryotoxicity assay as an alternative for higher vertebrates.

## 2. Materials and Methods

### 2.1. Search Strategy

A systematic review of scientific evidence of the use of the zebrafish embryotoxicity model for toxicity assessment of herbal medicine was undertaken in accordance with the PRISMA (Preferred Reporting Items for Systematic reviews and Meta-Analyses) [18, 19]. A comprehensive search of the literature was conducted in the following databases: PubMed (US National Library of Medicine, USA), SciVerse Scopus (Elsevier Properties SA, USA), and Google Scholar for studies published before 31 March 2019. The following medical subject headings and keywords, “Zebrafish embryo herbal medicine,” “Zebrafish herbal toxicity,” “Zebrafish embryo/larval herbal plant toxicity,” “Zebrafish embryo/larval toxicity medicinal plants,” were included in the search. Articles were selected based on the inclusion and exclusion criteria mentioned in Section 2.2.

All the papers obtained from searching the databases in compliance with the criteria were pooled together, and duplicates were removed. The remaining articles were initially screened by reading the “title” followed by reading the “abstracts.” Studies not satisfying the inclusion criteria were excluded at these stages. The remaining articles were screened in the final stage by reading the full text, and those not meeting inclusion criteria were excluded. The search process was conducted independently by two authors and the final group of articles.

### 2.2. Inclusion/Exclusion Criteria

The following inclusion criteria were applied for this study. Studies which have used the zebrafish embryos or larval stages to assess the toxicity of (a) crude herbal/medicinal preparation (b) different chemical fractions derived from herbal medicine, (c) group of compounds derived from herbal medicine or medicinal plants, (d) single compound extracted from herbal plants/medicine, and (e) herbal products/traditional prescriptions were included in this study.

Studies were excluded based on the following exclusion criteria: (a) assessment of toxicity use of juvenile or adult stages of the zebrafish, (b) studies which assessed the toxicity of other chemical compounds including synthetic compounds, (c) investigation of bioactivity of herbal medicine using zebrafish embryo other than toxicities, (d) studies investigating the protective effect of herbal medicine against toxicity induced in the zebrafish embryos by other chemicals, (e) studies conducted on herbal products which are used against dyes and preservatives, (f) articles written in other languages, (g) reviews, and (h) editorials: (i) conference proceedings and (j) commentaries.

## 3. Results and Discussion

**Figure 1:**
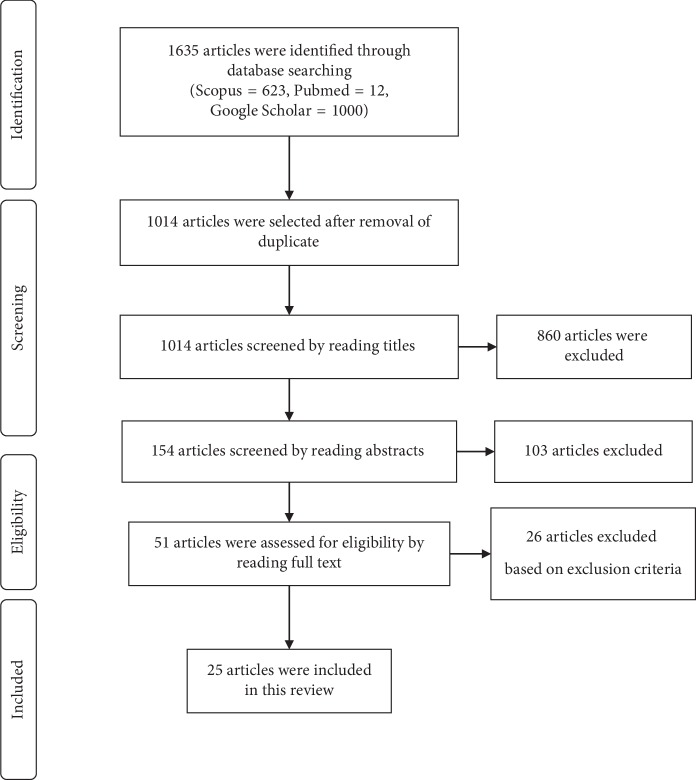
Search strategy followed to recruit the scientific evidence for the review.

The literature search identified the following number of articles in the respective databases: PubMed (*n* = 12), SciVerse Scopus (*n* = 623), and Google Scholar (*n* = 1000). After removing duplicates and excluding the paper based on the exclusion criteria, the total number of 25 articles were included in the present review.  Figure 1 presents the search strategy used in selecting the articles.

This systematic review pools the available scientific data and were categorized into three groups that have used the zebrafish embryotoxicity assay to investigate the toxicity of (1) herbal formulae/medical prescription (2 articles) (Table 1), (2) crude extracts (12 articles) (Tables 2, and (3) phytocompounds/isolated constituents (11 articles) (Table 3).

According to the available literature, thus far the number of herbal compounds investigated using this model is limited compared with other chemical or pollutants. During the literature survey, it was apparent that there are many conference proceedings related to the aforementioned topic. However, they were excluded from this review adhering to the exclusion criteria.

Medicinal plants have historically been invaluable as a source of therapeutic agents. In the last decade, herbal medicine has undergone some form of revival and advancing at a greater pace due to public acceptance as safe therapy [46]. Though herbal medicine is considered as safe, there are reported cases of adverse drug interactions, mutagenic, carcinogenic, and teratogenic effects [47]. Hence, there is a great concern for the toxicological evaluation of herbal medicine.

In the last three decades, there has been a tendency towards the limited use of higher animals in toxicological assays particularly in herbal toxicological studies [8]. The use of alternative assays or models that align with the 3R principal; reduce, refine, and replace for the use of higher vertebrates in toxicological experiments is encouraged [8].

Recently, zebrafish has emerged as an efficient animal model for *in vivo* high-content drug screening and toxicological analysis [47]. The overall predictive success rate of zebrafishes for drug-induced toxicity reached 100%, and it is ranked as an excellent model by the European Center for the Validation of Alternative Methods (ECVAM) [48]. Notably, zebrafish as whole organisms are apparently able to capture toxic chemicals/constituents which are unlikely to be found in *in vitro* cell culture systems [49].

According to this review, the zebrafish model has been employed to investigate the toxicity of 6 polyherbal formulae/medical prescriptions, 16 crude plant extracts, and more than 30 phytocompounds/isolated constituents. Thus, the results obtained from these studies are categorized into three groups.

### 3.1. Polyherbal Formulae/Medical Prescription

The zebrafish embryotoxicity model has been utilized to evaluate the toxicity of some common Chinese Medical Prescriptions (CMPs) and Chinese Patent Medicines (CPMs) (Table 1). These preparations are commercially available for the public and have become an important part of Traditional Chinese Medicine (TCMs). These prescriptions consist of several herbal/medicinal plants.

Si Jun Zi Tang (SJZT), Liu Jun Zi Tang (LJZT), and Shenling Baizhu San (SLBS) are CMPs commonly used to treat patients with poor appetite, loose stool, abdominal distension, lassitude, prolapsed anus, shortness of breath, dysphasia, and spontaneous sweating. Ding et al. [20] investigated the nephrotoxicity of the above-mentioned common CMPs (which includes several herbal plants) using the Transgenic zebrafish line *Tg* (*wt1b:EGFP*). In zebrafish, Tg line, the parts of the excretory system are labelled with a green fluorescent protein (GFP). The results indicated the survival rate of embryos significantly decreased in the highest concentration of all three CMP preparations (53.3 ± 8.1% to 86.7 ± 6.7%) at 4  hpf (hours post fertilization). A few defects in the kidneys of the embryos have been observed in 25 ng/mL concentration of each medical prescription. The percentage of kidney malformation phenotypes has increased with the increase in exposure concentrations (25 ng/mL, 0–10%; 250 ng/mL, 0–60%; and 1,250 ng/mL, 80–100%). It was revealed that these CMPs induced kidney malformation phenotypes and the effect was dose-dependent. Moreover, the kidneys of zebrafish embryos were more sensitive to SLBS than SJZT and LJZT [20].

Compound Danshen Tablet (CDT) (treatment for heart diseases), Angong Niuhuang Pill (ANP),(treatment for central nervous system diseases), and Lidan Paishi Tablet (LPT) (treatment for gallbladder diseases) are famous CPMs listed in Chinese Pharmacopeia. All of them have displayed teratogenic and lethal effects in zebrafish embryos. The LC_50_ values for CDT, ANP, and LPT were calculated as 417, 596, and 380 *μ*g/mL, respectively, for zebrafish embryos. Teratogenic effects were exerted by all three preparations with an EC_50_ value at 351, 793, and 220 *μ*g/mL for CDT, ANP, and LPT, respectively. Tail bending and pericardial oedema were displayed as main teratogenic effects in this experiment. Furthermore, CDT and LPT at high concentrations have induced heart rate reduction and showed potential cardiotoxicity [21].

### 3.2. Crude Plant Extracts

The zebrafish embryotoxicity model has been particularly advantageous in evaluating the toxicity of crude herbal preparation. This section includes several studies that have used the zebrafish embryo assay for toxicological assessment (Table 2). Around 16 crude plant extracts have been subjected to zebrafish embryotoxicity and some of the studies have validated these results using other toxicity models such as mouse/rat models or computation methods.

*Andrographis paniculata,* (AP) (leaves), *Cinnamon* zeylanicum (CZ) (bark), C. xanthorrhiza (CX) (rhizome), E. polyantha (EP) (leaves), and O. stamineus (OS) (whole plant) are popular medicinal plants used in Asian regions. These plants are popular to contain strong antioxidant. This study has used the zebrafish embryos to assess the toxicities of the water extracts of the aforementioned plants. The results revealed the bark extract of CZ exhibited the highest toxicity with LC_50_ value of 0.0508 mg/mL, followed by leaves of EP (0.06039 mg/mL), leaves of AP (0.5256 mg/mL), rhizome of CX (0.7037 mg/mL), and whole plant of OS (1.685 mg/mL). Teratogenic defects such as the bent spine, enlarged yolk sac, pericardial oedema, slow heartbeat, and delayed hatching (>72 hpf) were also reported. Thus, out of all plant's the whole plant extract of OS was found as the lowest toxic to zebrafish embryos [22]. The toxic effects of these plant extracts were compared using *in vitro* cytotoxicity assay using 1.1B4 (human-derived pancreatic *β*-cell line), 3T3-L1 (mouse-derived adipocyte like cells), and WRL-68 cell (human hepatic cell line) types. The results predicted that the LC_50_ at 48 hpf and IC_50_-3T3-L1 correlates well [22].

*Tinospora cordifolia*, commonly known as “*makabuhay”*, is a medicinal plant in the Philippines which is widely used as antibacterial, analgesic, and antipyretic agent. It is also used for the treatment of jaundice, skin diseases, and anaemia. The water extracts of bark and leaves of this plant were tested on zebrafish embryos to evaluate the toxic effects. Both bark and leaf extracts indicated dose-dependent and time-dependent toxicities against the zebrafish embryos. Among the two extracts, 5% and 10% of leaf extract recorded the highest mortality of 100%. However, the bark extract showed a mortality of 11.11% and 33.33% at 5% and 10% concentrations, respectively. In 5% or higher concentrations of leaf extract and in 10% of bark extract, none of the embryos was hatched. The heartbeat rates of the zebrafish embryos that exposed to both bark and leaf extracts were significantly decreased. Furthermore, different teratogenic effects such as head and tail malformations, delayed growth, limited movement, scoliosis/flexure, and stunted tail were reported with dose-dependance and plant parts-dependence. This study showed both leaf and bark extracts of the *T. cordifolia* were toxic to the zebrafish embryos and leaf extract was found to be more toxic [23]. Generally, cardiac glycoside in plant material is one of the most commonly associated compounds that affect cardiac functions. The cardiotoxic effect of *T. cordifolia* can be governed by cardiac glycoside present as the active component [23].

The zebrafish embryotoxicity model has been employed to evaluate the acute toxicity of ethanolic extract of pomegranate (*Punica granatum* L) peel. Pomegranate peel is prescribed as an antimicrobial agent. The ethanol extract of pomegranate peel revealed LC_50_ of 196.037 ± 9.2 *μ*g/mL exposure of the zebrafish embryos for 96 hours indicating that it is safe against zebrafish embryos [24]. This study has also used ADMET Predictor 7.1 program for prediction of toxicity of the compounds found in the peel of pomegranate and agrees with zebrafish assay that there is no compound that is toxic to reproductive organs, heart, and androgen hormones [24].

*Geissospermum reticulatum* A. Gentry is a tree commonly found in the Amazon. Traditionally, various parts of this tree are known to possess antimalarial, antitumoral, antioxidant, nociceptive, and antibacterial activities. The zebrafish embryotoxicity assay performed for the ethanolic extract of the bark of *G reticulatum* revealed even at the highest concentration tested was nontoxic to the zebrafish embryos. Embryo deaths, deformities, and teratogenic effects were not reported in this study signifying the safety of the extract [25]. However, contrary to the results, the ethanolic extract of the bark of *G reticulatum* exhibited cytotoxicity in the *in vitro* tests on malignant cells THP-1 (human monocytic cell line) and HL-60 cells (human leukemia cell line) [25].

The zebrafish embryotoxicity assay has been employed to investigate the toxic effect of *Curcuma longa*. It is popular as traditional medicinal preparations and in everyday culinary. Studies have validated several medicinal properties such as antioxidant activity, cardiovascular and antidiabetic effects, inflammatory and edematic disorders, anticancer, antimicrobial, hepatoprotection, and protection against Alzheimer's and photo protector. Several studies have been conducted to test the toxicity of *C. longa* in which the zebrafish embryotoxicity model was employed. The methanol extract of the *C. longa* dosage at 62.5 *μ*g/mL exhibited teratogenic effect and higher concentrations caused physical body deformities such as kink tail, bend trunk, and enlarged yolk sac. At 96 and 120 hpf, kink and bend tail were observed, respectively, in embryos treated with 62.5 *μ*g/mL. Dosage at 125.0 *μ*g/mL, resulted in embryo mortality and detected physical body deformities of larvae among the hatched embryos. The LC_50_ value for 24, 48,72, 96, and 120 hrs was calculated as 92.41, 79.19, 68.31, 56.67, and 55.89 *μ*g/mL, respectively [26].

Safflower (*Carthamus tinctorius* L.) is a medicinal plant grown in China which is mainly used in the treatment of blood stasis syndrome with dysmenorrhea, amenorrhea, postpartum abdominal pain and mass, and trauma and pain in the joints. However, this herb is known to induce abortion. Hence, the zebrafish assay was employed to assess the development toxicities of flowers of safflowers. Safflower delayed the hatching and caused abnormal spontaneous movement reporting depressed heart rate, pericardial oedema, yolk-sac oedema, abnormal head-trunk angle, inhibition of melanin release, enlarged yolk, and short body length. The 96 h LC_50_ of safflower to zebrafish embryos was reported as 345.6 mg/L. Heart rate is a crucial measurement reflecting cardiac developmental toxicity. Concentration at 250 mg/L revealed significant inhibition of heartbeat at 48 hpf and 72 hpf. The heart rate has been decreased progressively with increasing concentrations [27]. The molecular mechanism underlying the toxic effect of safflower was correlated with the altered activities of defense enzymes (SOD, CAT, and GPX), increased content of MDA, decreased caspase-3 activity, and altered the mRNA levels of related genes in zebrafish larvae. Hence, it is inferred that the oxidative stress and increased apoptosis is responsible for development abnormalities in zebrafish following the exposure of safflower [27].

Fuzi is the lateral root of *Aconitum carmichaeli* which is prescribed in traditional Chinese medicine as a remedy against cardiotonic, analgesic, anti-inflammatory, and diuretic agents to treat colds, polyarthralgia, diarrhea, heart failure, beriberi, and pericardial oedema. Three types of preparations, namely, FZ-0 (water suspension of powdered material), FZ-60 (60-minute decoction), and FZ-120 (120 min decoction) were tested for toxicity on zebrafish embryos. Based on the toxicity experiment conducted with mice, it was indicated that FZ-120 is less toxic; hence, FZ-120 decoction was further tested on the zebrafish embryotoxicity model. The results revealed FZ-120 caused the death of zebrafish embryos from 700 to above 1000 *μ*g/mL concentrations. Abnormalities were observed in the heart, liver, yolk sac, swim bladder, and body length mainly at doses ranging from 288 to 896 *μ*g/mL of FZ-120. This study highlighted that the long-term decoction is not adequate for detoxification of Fuzi [28]. Furthermore, this highlighted that the toxicity of FZ-120 was higher than other decoctions, revealed even with the rodent model [28]. Moreover, this study utilized the UPLC-MS assay (Ultra Performance Liquid Chromatography-Mass Spectrometry) to identify the toxic compounds in the FZ-120 and suggested an appropriate test system to unravel aconitine-related acute toxicity [28].

*Carpesii Fructus*, the dried fruit of *Carpesium abrotanoides* L, is a traditional Chinese remedy used to cure the intestinal worms in children. The zebrafish embryotoxicity revealed the LC_50_ value of *Carpesii Fructus* as 230.40 mg/L. Some developmental abnormalities such as hatching inhibition, increased spontaneous movement, heartbeat inhibition, pericardial oedema, yolk-sac oedema, bleeding tendency, yolk malformation, enlarged yolk, and shortened body length were observed in this study [29]. In depth molecular studies highlighted that the changed activities of defense enzymes, increased malondialdehyde (MDA) content, decreased caspase-3 activity, and altered mRNA levels of oxdative stress-related genes (ogg1, p53, Cu/Zn-Sod, Mn-Sod, and Cat; Gpx) in zebrafish larvae playing a major role in developmental toxicities caused by the *C. Fructus* [29].

*Sutherlandia frutescens* (L.) R.Br is a valuable medicinal plant in South Africa. This plant is widely used against asthma, dysentery, fever, gastritis, diabetes, and as an immune boost. Both ethanol and water extracts of the plant were tested on zebrafish embryos. The toxicity assessment revealed some chronic teratogenic toxicities, leading to pericardial oedema, yolk sac swelling, and other abnormal developmental characteristics. Higher doses of the ethanolic extract were indicated the greater incidence of aberrant morphological formations, recorded at a frequency of 38% when embryos were exposed to a 200 *μ*g/mL extract. A treatment of 300 *μ*g/mL with both extracts (the highest concentration) resulted in lethal toxicity, and no embryo was hatched at this concentration [30].

*Leonurus japonicus Houtt*. (motherwort) is a Chinese medicine. The aerial parts of this plant are prescribed against gynaecological and obstetrical conditions, such as menstrual blood stasis, menstrual disturbances, dysmenorrhea, amenorrhea, postpartum haemorrhage, and postpartum recovery. The zebrafish embryos were treated with motherwort essential oil at 2 hpf, 10 hpf, 24 hpf, and 48 hpf, respectively. The LC_50_ of zebrafish embryos treated at 2 hpf, 10 hpf, and 24 hpf (around 10 *μ*g/mL) were much lower than those of zebrafish embryos treated at 48 hpf (around 60 *μ*g/mL), indicating early stages are more sensitive to motherwort essential oil. Similarly, the teratogenic effects of embryos treated with motherwort essential oil also indicated the early stages are much sensitive. The TC_50_ (teratogenic effect) of 2 hpf embryos was much lower (1.67 ± 0.23 *μ*g/mL) compared with that of 10 hpf and 24 hpf (TC_50_-10 *μ*g/mL) and 48 hpf (TC_50_-20 *μ*g/mL).

Zebrafish embryos also exhibit abnormalities in spine together with yolk-sac oedema. The average embryonic heart rate was also decreased in embryos exposed to 25, 50, or 100 *μ*g/mL. [31].

Radix *Sophorae tonkinensis* (RST) is a Chinese medicine used to cure infectious and inflammatory diseases. The whole RST extract and its ethanol sediment (RSTE) and its active fractions prepared using five different types of solvents, dealkalized water, ethanol, *n*-butyl ethanol, dichloromethane, and diethyl ether were tested for toxicity using the zebrafish embryotoxicity model. Concentration-dependent mortality was demonstrated by RSTE, RST, and active fractions in zebrafish embryos. Pericardial oedema and/or reduced heart rates were observed in the zebrafish treated with dichloromethane extract, *n*-butyl ethanol extract, and diethyl ether extract in a dose-dependent manner, but was not observed with RSTE, dealkalized water extract, or ethanol sedimentation extract. Cardiovascular toxicity was observed for the fraction extracted using diethyl ether, while hepatotoxicity was observed for the whole RST extract and the fractions extracted using water and ethanol. Both cardiovascular and hepatic toxicities were observed for the fractions extracted using *n*-butyl ethanol and dichloromethane [32].

The dried root of *Euphorbia kansui* is a traditional Chinese medicine used against cancer, pancreatitis, and intestinal obstruction. Clinical application of this is restricted due to severe toxicity caused by *Euphorbia kansui*. The present study has attempted to treat the *E. kansui* with vinegar to reduce the toxicity. The ethyl acetate extract which was extracted from *Euphorbia kansui* (KS-1) and *Euphorbia kansui* fry-baked with vinegar (KS-2) were tested on zebrafish embryos for toxicity. The LC_50_ value for *Euphorbia kansui* (KS-1) and fry-baked with vinegar KS-2 was reported as 2.78 ± 0.86 *μ*g/mL and 6.62 ± 1.24 *μ*g/mL, respectively, indicating that the KS-2 was less toxic than KS-1. In addition to the mortality, the teratogenic effects such as pericardial oedema and scoliosis were reported in embryos treated with both preparations [33]. This study provides the pieces of evidence for the reduced toxicity of *E. kansui* treated with vinegar [33].

### 3.3. Phytocompounds/Isolated Constituents

According to the literature, the zebrafish model has been employed to investigate the toxicity of isolated phytocompounds and constituents from herbal medicine (Table 3).

*Polygonum multiflorum Thunb*., is a traditional medicinal plant which has been used widely in East Asia. This plant is commonly used as an antiageing, antihyperlipidaemic, antioxidant, anti-inflammatory, anticancer, hepatoprotective, and immunomodulatory agent. The zebrafish embryotoxicity assay has been employed to assess the toxicity of different extractions, compounds, and constituents. Toxicity order of the different extracting solvent on the zebrafish has been reported as 70% ethanol >95% ethanol >50% ethanol ≅ methanol >30% ethanol > acetone > water. Four components were isolated from 70% ethanol fraction where the toxicity of component (D) was found to be higher than that of the other components. Study on the chemical constituents of component D revealed the presence of 27 compounds, including 7 anthraquinones (1–7), 8 stilbenes (8–15), 7 anthrones (16–22), 3 cinnamic acid amides (23–25), and 2 naphthols (26–27), and they were isolated and assessed in zebrafish embryos. Out of them, five anthraquinones, seven anthrones, and two naphthols showed obvious toxicity which suggested that these compounds may be the potentially toxic components in *P. multiflorum* [30]. LD_50_ was calculated for each compound [34].

Matrine and sophocarpine are two alkaloids found in the root of Sophora flavescens commonly known as Kushen in traditional Chinese medicine (TCM). These two matrine-type alkaloids exhibited a variety of pharmacological properties such as anti-inflammatory, antiviral, antitumour, and antiarrhythmic activities. When the zebrafish embryotoxicity model was employed to test the toxicity of these compounds, both alkaloids displayed teratogenic and lethal effects with the EC_50_ and LC_50_ values at 145 and 240 mg/L for matrine and 87.1 and 166 mg/L for sophocarpine, respectively. Teratogenetic effects such as pericardial oedema, tail malformation, notochord malformation, scoliosis, yolk oedema, and growth retardation were observed after 48 hrs. Furthermore, these alkaloids significantly altered spontaneous movement and inhibited swimming performance of larvae at concentrations below that caused the embryo mortality and malformations, indicating a neurotoxic potential of both drugs [35].

Celastrol is a terpenoid purified from *Tripterygium wilfordii* Hook F. This compound possesses antioxidant and anti-inflammatory potential, and it is recommended against neurodegenerative disorders and cancer. The toxicity studies carried out using the zebrafish embryotoxicity model revealed hatching rates of embryos treated with 1.0 *μ*M or higher concentrations of celastrol were significantly lower than that of the control. The lethal effect of celastrol on zebrafish embryos was dose-dependent, and the LC_50_ value of celastrol on embryos was approximately 1.40 *μ*M. Several developmental abnormalities, including no blood flow, oedema in the pericardial sac, and tail malformation were reported in embryos treated with 0.5 *μ*M or higher concentrations of celastrol. Particularly for tail malformation, the EC_50_ was calculated as 0.66 *μ*M at 72 hpf [36].

Emodin is an anthraquinone derivative; it is the main effective monomer of herbal rhubarb. It is a natural pigment found in the root and bark of many plants of the genus *Rhamnus*. It has a variety of pharmacological actions such as antidiabetic, antinociception, anticancer, and cholesterol reduction potential. The zebrafish at 7 days after fertilization were exposed to a series of concentrations ranging from 0.1, 0.25, 0.5, 0.75, 1, 1.5, and 2 *μ*g/mL to assess the toxicity. Emodin exhibited a dose-related increase in mortality, with significant death of embryos at a drug threshold of 0.25 *μ*g/mL. The LD_50_ value (at 72 hpf) of emodin on 7 hpf embryos were approximately 0.20 *μ*g/mL. Malformations such as oedema, crooked trunk, and abnormal morphogenesis of some organs, such as statolith, swimming bladder, and yolk syncytium, were reported in embryo treated with 0.1–1.5 *μ*g/mL emodin [37]. In depth investigation of the molecular mechanism revealed that there is an increase in mRNA accumulation of drug-metabolism genes (CYP3A) and a multiple drug-resistance gene (MDR1) in embryos. Also, the emodin-related impairment is related to the expression of these genes [33]. An ortholog of CYP3A in zebrafish was homologous to the human CYP3A subfamily, which was initially transcribed only in the liver and intestine upon hatching of the zebrafish. According to the study, both genes were upregulated in zebrafish embryos following the treatment with emodin [37].

Cannabidiol (CBD) is an active compound found in *Cannabis sativa.* It is a nonpsychotomimetic compound that has been used for the treatment of severe neuropsychiatric disorders. The zebrafish embryos exposed to CBD (all concentrations) did not show significant morphological malformations. However, the CBD at the highest concentration (300 *μ*g/L) has significantly delayed the hatching time of embryos. Moreover, embryos exposed to CBD did not show differences in the acetylcholinesterase activity, but embryos exposed to CBD 20–300 *μ*g/L were 1.4 up to 1.7-fold more active when compared with the control indicating a CBD modulates the motor activities [34]. Surprisingly, CBD did not display significant alterations in acetyl chlorine esterase (AChE) in concentrations correlated with human plasma dose [34]. Secondarily, CBD was tested on motor systems [38] as motor responses of zebrafish are highly sensitive to the neurotoxic chemical compound which is associated with cannabinoid receptors-mediated (CB1) regulation. It was shown that CBD increased the motor activities of zebrafish embryos after 24 hpf, acting as an antagonist of the CB1 receptor [38].

Aristolochic acid (AA) (nitrophenanthrene carboxylic acid) is found primarily in *Aristolochia* or *Asarum*. The extracts containing AA have been used in medical therapies for arthritis, gout, and festering wounds. The zebrafish embryotoxicity model was used to evaluate the nephrotoxicity, highlighting the importance of nephrotoxicity investigations as a crucial aspect of herbal toxicities. There was a significant difference in the survival rate of the embryos between the treated and control experiments. AA-treated (10 ppm) embryos exhibited significantly reduced glomerular filtration rates (GFRs) (at 3–5 h; 71.48 ± 18.84%∼39.41 ± 15.88%) compared with the control (100 ± 2.24%). Furthermore, AA-treated zebrafish embryos displayed malformed kidney phenotypes, such as curved, cystic pronephric tubes, pronephric ducts, and cases of atrophic glomeruli. The percentages of embryos with malformed kidney phenotypes increased as the exposure dosages of AA increased. AA-treated zebrafish embryos also exhibited deformed hearts, swollen pericardium, impaired blood circulation, and the accumulation(s) of red blood cells [39].

Psoralen is an active compound found in *Psoralea corylifolia* L., a Chinese herb, which is widely used in traditional medicine for the treatment of psoriasis, vitiligo, osteoporosis, osteosarcoma, bone fracture, and osteomalacia. Zebrafish AB strain and the *Tg(cmlc2:EGFP), Tg(L-FABP:EGFP), Tg(Lyz:EGFP),* and *Tg(Vmat:GFP)* transgenic zebrafish lines were used for lethality and other specific organ toxicity studies. Mortality rates in the treatment group exhibited a dose and time-dependent increase. The values of LC_50_, LC_10_, and LC_1_ at 96 hpf were determined to be 18.24, 13.54, and 10.61 *μ*M, respectively. The 13.54 *μ*M psoralen-treated group exhibited lower hatching rate (70%) than that of the control group (94%). Several morphological abnormalities such as the yolk retention, swim-bladder deficiency, pericardial oedema, and curved body shape were observed at 24 to 96 hpf in psoralen-treated embryos. Also, it was observed that psoralen exerted toxic effects on the developing heart, liver, phagocytes, and nervous system [40]. Increased generation of reactive oxygen species and malondialdehyde concentrations and inhibition of total superoxide dismutase activity in the zebrafish embryo indicate oxidative stress caused by the psoralen [40].

The zebrafish embryotoxicity assay was used to evaluate the toxicity of bioactive fraction of the Korean medicinal plant *Artemisia capillaries* Thunberg. The novel compound isofraxidin 7-O-(6′-*O*-*p*-coumaroyl)-*β*-glucopyranoside (compound 1) was isolated and is known to enhance the pigmentation. It is believed that this compound can be developed as a pharmaceutical/cosmetic agent to treat skin disorders resulting from reduced pigmentation. The toxicity indicators, mortality rate, and heart rate in treated zebrafish embryos to identify the safe and effective concentration of compound 1. Interestingly, the compound 1 caused no mortality in treated embryos. Greater than 90% of the treated embryos survived, which was not significantly different from the control group. The results revealed that the compound 1-(25 *μ*M) treated embryos showed no developmental defects and displayed normal cardiac function, indicating that this compound enhanced pigmentation without producing toxicity [41].

Evodiamine is a bioactive alkaloid found in *Evodia rutaecara*, a Chinese medicinal plant. It is recommended for abdominal pain, headache, menstrual problems, vomiting, and diarrhea. The concentration of 50–100 ng/mL evodiamine exposure for 24 hrs caused no lethal effects. However, concentrations ≥400 ng/mL significantly increased, and the lethality reached 100% at 1600 ng/mL evodiamine. The atrial and ventricular heart rates were decreased in a dose-dependent manner in zebrafish exposed to evodiamine. Moreover, 354 ng/mL concentration of evodiamine has induced cardiac malfunction, as evidenced by changes in heart rate and circulation, and pericardial malformations [42]. The present study investigated the effects of evodiamine on primary cultured neonatal rat cardiomyocytes *in vitro*, and both studies indicate potential cardiotoxic effects of evodiamine [42].

Tanshinone IIA (Tan-IIA) is a diterpene quinone derived from the dried roots of *Salvia miltiorrhiza Bunge*, a traditional Chinese medicine. The compound is commonly used against cardiovascular disease and exhibits various pharmacological activities, including anti-inflammatory, antioxidative, antifibrosis, modulation of collagen metabolism, and antitumour. Both normal and dechorionated zebrafish embryos were used to assess the developmental and cardiotoxicity caused by Tan-IIA. The LC_50_ values in the dechorionated embryo group at 72 hpf and 96 hpf were calculated as 18.5 *μ*M and 12.8 *μ*M, respectively. Teratogenic effects such as scoliosis, malformation of the tail, and pericardium oedema of dechorionated embryos were manifested at a concentration of about 1 *μ*M. Normal embryos were less sensitive to Tan-IIA, but with the increasing concentration, severe abnormalities of heart and pericardium were observed. Moreover, after treatment with Tan-IIA ≥ 6 *μ*M, the time it took for the embryos to mature into fish was longer than that for the control [43].

Gambogic acid (GA) is an active ingredient of gamboge extracted from the dried yellow resin of *Garcinia hanburyi Hook*.f., which is traditionally claimed against cancer. Gambogic acid treatment caused a pectoral fin defect and lethal toxicity in zebrafish embryos in a dose-dependent manner. The LC_50_, LC_10_, MNLC (maximum nonlethal concentration), and EC_50_ values were calculated as 1.76 *μ*M, 0.8 *μ*M, 0.5 *μ*M, and 0.723 *μ*M, respectively. GA at 0.5–1.0 *μ*M exhibited specific fin developmental defect with the phenotype resembling those caused by thalidomide. The amount of GA absorbed by the zebrafish embryos appeared to be time dependent [40]. It was further revealed GA upregulated ALDH12 (Aldehyde Dehydrogenase 1 Family Member A2) and downregulated CYP26A1 (cytochrome P450 family 26 subfamily A member 1) at 8hpf. These genes are the target of RA signaling (retinoic acid signaling) that disturb the development of the pectoral fin [44].

Diterpene alkaloids (DAs) are phytochemicals possessing significant pharmacological properties such as anti-inflammatory, antidepressant, antiarrhythmic, antiplatelet aggregation, and antimalarial properties. Despite the pharmacological importance, there is a concern of cardiotoxicity of DAs. Thus, the heart-specific green fluorescence zebrafish model was introduced for evaluating the cardiotoxicity. This study tested the toxicity of three DAs, namely, Aconitine (AC), Mesaconitine (MAC,) and Hypaconitine (HAC) and three Monoester diterpene alkaloids (MDAs),namely, 14-a-benzoylaconine (BAC), 14-a-Benzoylmesaconine (BMAC), and Benzoylhypaconine (BHAC), particularly for cardiotoxicity. The heart rate of the embryos was decreased by the AC, MAC, and HAC at low doses (15.6 and 31.3 *μ*M) and was increased at high doses (62.5, 125, and 250 *μ*M). On the other hand, BAC, BMAC, and BHAC decreased the heart rates in the dose range of 31.3–250 *μ*M, while the highest dose (500 *μ*M) of BAC and BMAC increased the heart rates. In addition, the diterpenes including AC, MAC, and HAC exhibited serious organic and functional toxicities in zebrafish embryos compared with monoester diterpene alkaloid [41]. The toxicity is attributed to the presence of compounds that activate the Na+ channel K+ channel inhibitors, respectively. These Na+ channels were believed to activate to increase the heart rate. Then, the increase in heart rate made the myocardium overloaded and finally resulted in functional and organic damage on the zebrafish heart [45].

## 4. Conclusion

The information gathered in this review collectively indicates the zebrafish embryotoxicity model holds a great promise in herbal toxicity assessment and provides an ideal alternative to laboratory animals like rats, mice, or rabbits. Zebrafish embryotoxicity assessment is in compliance with the 3R' principal of animal welfare and proceeds through a holistic screening approach of herbal medicine to reach clinical conclusions. Low cost, easy handling, the requirement of small quantities of test compounds, and high throughput screening make it a highly competitive and successful model to be used in toxicity studies. Most importantly, early life stages of zebrafish are considered as less pain or discomfort when exposed to chemicals. Hence, this model has become a popular alternative for higher animals, used in herbal toxicity assessment.

Despite the popularity as an alternative toxicological model, thus far the number of herbal compounds investigated using this model is limited compared to other chemical compounds. During the literature survey, it was apparent that there are many conference proceedings related to the current topic. However, they were excluded from this review adhering to the exclusion criteria.

According to this review, the zebrafish model has been employed to investigate the toxicity of 6 polyherbal formulae/medical prescriptions, 16 crude plant extracts, and more than 30 phytocompounds/isolated constituents. Generally, TCMs were regarded as less toxic with no contraindication in pregnancy. However, the studies entailed in this review revealed embryo and development toxicities of TMCs. Thus, it is prudent to test the toxicological impact of TCMs using the zebrafish embryotoxicity model as a high throughput screening prior to confirming their safety for human consumption.

Majority of the studies included in this review investigated the toxicity of crude plant extracts or isolated compounds. Hence, it is inferred that this model is more applicable for single compounds and toxicity prediction, i.e., straight forward. However, when analyzing the toxicity of crude extracts, this model appeared to couple with analytical methods such as the HPLC or UPLC-MS assay to identify the individual toxic compound.

Most of the studies included in this review used the zebrafish embryo model to explicate the lethality and development toxicities. The LC_50_ values were calculated based on the lethality endpoint, coagulation, lack of somite formation, lack of heartbeat, and none detachment of tail end compliance with the OECD guidelines. The EC_50_ values were calculated based on teratogenic effects. Most studies entailed here followed OECD test protocols to elucidate the toxicity of herbal medicine.

According to the review, the zebrafish model was applied to investigate specific toxicities such as nephro, cardio, and neurotoxicities. Particularly, the zebrafish model was effective in detecting nephrotoxicity. The green fluorescence kidney transgenic zebrafish provides a good model over mice and human to detect kidney malformation caused by chemicals such as aristolochic acid (AA) in medicinal plants. Similarly, the zebrafish model provides valuable information about cardiotoxicity. The heart-specific green fluorescence zebrafish lines are available now to investigate the specific toxic effect on the heart. The plant extracts with cardiac glycoside which cause the cardiotoxicity can be easily detected. Furthermore, neurotoxic potential of herbal medicine was also evaluated with the zebrafish embryo toxic model as exemplified in cannabis extracts.

Few studies included in this review used other biological models such as rat or mice model or *in vitro* cytotoxicity assays to compare the toxicological effect. Some studies revealed that cytotoxic potencies of fish and mammalian cell lines were almost equally sensitive while some showed controversial results. It was suggested that the cytotoxic substance(s) that may not be taken up by the zebrafish embryos or specific signaling pathways that causes cytotoxicity may be absent in zebrafish embryos. Moreover, there are some important differences between the zebrafish and mammals, such as the ectothermic nature of zebrafish with no cardiac septa, synovial joints, and lungs. Thus, some phenotypic effects produced by zebrafish are difficult to extrapolate to humans. Furthermore, there is no reliable method of dose translation from zebrafish to human.

Furthermore, this model was instrumental in investigating the underlying molecular mechanisms. Zebrafish embryos are more easily subjected to molecular screening such as analysis of gene expression studies. Numerous studies applied this model to investigate the teratogenic effects due to disruption of the oxidative/antioxidant enzyme (SOD, Catalase, and GPX) system by constituents in plant extracts. Moreover, this model provides an effective detection of the activities of some specific genes such as drug-metabolism genes (CYP3A) and a multiple drug-resistance gene (MDR1) which can be altered by phytocompounds like emodin. Hence, this model will undoubtedly bring about significant advances in predicting toxicity of herbal medicine.

This review provides ample affirmation of applicability of the zebrafish embryotoxicity model for herbal toxicology. It is anticipated, in future, the zebrafish model may provide a novel vista in herbal toxicology as an alternative assay to higher vertebrates.

## Figures and Tables

**Table 1:** Evaluation of toxicity of herbal formulae/prescriptions using the Zebrafish embryotoxicity model.

No	Herb formulae/medical prescription	Medicinal use	Type of zebrafish	Toxic effects		References	Toxicity compared with other assays
				Survival/mortality	Teratogenic and other toxic effects		
1	Chinese medical prescriptions (CMPs) Si jun Zi Tang (SJZT) Liu jun Zi Tang (LJZT) Shenling Baizhu (SLBS)	Prescribed for poor appetite, loose stool, abdominal distension, lassitude, prolapsed anus, shortness of breath, dysphasia, and spontaneous sweating	Transgenic zebrafish line *Tg* (*wt1b:EGFP*)	All three CMPs exhibited 93.3 ± 6.7% to 100 ± 0.0% of survival at 48 hpf after exposure to 25 and 250 ng/mL. The survival rates decreased to 53.3 ± 8.1% to 86.7 ± 6.7% when exposed to 1250 ng/mL of all CMPs	The % of kidney malformation reported for different concentration 25 ng/mL, 0–10%; 250 ng/mL, 0–60%; 1,250 ng/mL, 80–100%) Embryonic zebrafish kidney was more sensitive to SLBS	[20]	Not compared

2	Chinese patent medicine (CPM), compound Danshen Tablet (CDT), Angong Niuhuang Pill (ANP), and Lidan Paishi Tablet (LPT)	Used to treat heart diseases, central nervous system diseases, and gallbladder diseases	AB strain zebrafish	The LC_50_ values for CDT, ANP, and LPT were calculated as 417, 596, and 380 *μ*g/mL, respectively.	EC_50_ values of teratogenic effects were 351, 793, and 220 *μ*g/mL for CDT, ANP, and LPT. The tail bending and cardiac oedema were the main teratogenic effects. CDT and LPT were cardiotoxic	[21]	Not compared

**Table 2:** Evaluation of toxicity of crude preparations and active fractions of herbal medicine using the Zebrafish embryotoxicity model.

No	Medicinal/herbal plant			Medicinal value	Toxic effects		References	Toxicity compared with other assays
	Scientific name	Common name	Part of the plant		Survival/mortality rate	Teratogenic and other toxic effects		
1	*Andrographis paniculata*	Green chireta	Leaves	Recommended for various illness antioxidant Potentials	LC_50_: 0.52 mg/mL (48 hrs) LC_50_: 0.52 mg/mL (96 hrs)	Teratogenic effect such as abnormal organ development demonstrated bent spine, enlarged yolk sac, pericardial oedema, slow heartbeat, and delayed hatching (>72 hpf)	[22]	LC_50_: 48 hpf and IC_50_: 3T3-L1 was the closest correlation.
2	*Curcuma xanthorrhiza*	Temulawak, java ginger	Rhizome		LC_50_: 0.74 mg/mL (48 hrs) LC_50_: 0.70 mg/mL (96 hrs)			
3	*Cinnamon zeylanicum*	Cinnamon	Bark		LC_50_: 0.98 mg/mL (48 hrs) LC_50_: 0.051 mg/mL (96 hrs)			
4	*Eugenia polyantha*	Indian bay leaf and Indonesian bay leaf	Leaves		LC_50_: 0.92 mg/mL (48 hrs) LC_50_: 0.06 mg/mL (96 hrs)			
5	*Orthosiphon stamineus*	Java or Cat's whiskers	Whole plant		LC_50_: 1.68 mg/mL (48 hrs) LC_50_: 1.68 mg/mL (96 hrs)			
6	*Tinospora cordifolia*,	Makabuhay	Leaves and bark	Antibacterial, analgesic, antipyretic, and also for the treatment of jaundice, skin diseases, and anaemia	5% and 10% of leaf extracts exhibited the highest mortality of 100%. Bark extract showed mortality of 11.11% and 33.33% at 5% and 10% concentrations	Head and tail malformations, delayed growth, limited movement, scoliosis/flexure, and stunted tail and these are dose- and plant parts-dependent. Leaf extract is more toxic than bark	[23]	Not compared
7	*Punica granatum* L	Pomegranate	Peel	Antimicrobial	LC_50_ of 196,037 ± 9,2 *µ*g/mL (96 hrs) considered as safe	No teratogenic and other effects	[24]	ADMET Predictor 7.1 program.
8	*Geissospermum reticulatum* a		Bark	Antimalarial, antitumoral, antioxidant, nociceptive, and antibacterial activities	Did not cause any visible death	Deformation or teratogenic effect were not observed	[25]	THP-1 and HL: 60 cells cytotoxic
9	*Curcuma longa*	Turmeric	Rhizome	Antioxidant activity, cardiovascular and antidiabetic effects, inflammatory and edematic disorders, anticancer, antimicrobial, and hepatoprotection	The LC_50_ values for 24, 48, 72, 96, and 120 are 92.41, 79.19, 68.31, 56.67, and 55.89 *μ*g/mL, respectively	Dosage at 62.5 *μ*g/mL indicated teratogenic effect of the extract was severe at higher concentrations producing physical body deformities such as kink tail, bend trunk, and enlarged yolk-sac oedema	[26]	Not compared
10	*Carthamus tinctorius* L.	Safflower	Flowers	Blood stasis syndrome with dysmenorrhea, amenorrhea, postpartum abdominal pain and mass, and trauma and pain in the joints	The 96 h LC_50_ of safflower to zebrafish embryos was reported as 345.6 mg/L Delayed hatching was reported	Abnormal spontaneous movement, depressed heart rate, pericardial oedema, yolk-sac oedema, abnormal head-trunk angle, inhibition of melanin release, enlarged yolk, short body length, and significant inhibition of heartbeat	[27]	Not compared
11	*Aconitum carmichaeli* Debx.	Fuzi	Lateral root	Cardiotonic, analgesic, anti-inflammatory, and diuretic agents to treat colds, polyarthralgia, diarrhea, heart failure, beriberi, and oedema	FZ-120 caused the death of zebrafish from 700 to above 1000 *μ*g/mL indicating potential toxicity	Abnormalities of heart, liver, yolk sac, swim bladder, and body length mainly at doses ranging from 288 to 896 *μ*g/ml	[28]	Acute toxic effect of mice. Similar results were observed
12	*Carpesium abrotanoides* L	Carpesii fructus	Dried fruit	Used against intestinal worms in children	LC_50_ value of *Carpesii Fructus* as 230.40 mg/L	Increased spontaneous movement, heartbeat inhibition, pericardial oedema, yolk-sac oedema, bleeding tendency, yolk malformation, enlarged yolk, and shortened body length	[29]	Not compared
13	*Sutherlandia frutescens*		Whole plant	Used for asthma, dysentery, fever, gastritis, diabetes; immune boost	A treatment of 300 *μ*g/mL with both extracts The highest concentration, resulted in acute lethal toxicity and no embryo was hatched at this concentration	Chronic teratogenic toxicities, leading to pericardial oedema, yolk-sac swelling, and other abnormal developmental characteristics	[30]	Not compared
14	*Leonurus japonicus Houtt*.	Motherwort	Essential oil	Against gynaecological and obstetrical conditions, such as menstrual blood stasis, menstrual disturbances, dysmenorrhea, amenorrhea, postpartum haemorrhage, and postpartum recovery	The LC_50_ of zebrafish embryos treated at 2 hpf, 10 hpf, and 24 hpf (around 10 *μ*g/mL) were much lower than those of zebrafish embryos treated at 48 hpf (around 60 μg/mL), indicating early stages are more sensitive to motherwort essential oil	The TC_50_ (teratogenic effect) of 2 hpf embryos was much lower (1.67 ± 0.23 *μ*g/mL) compared to that of 10 hpf and 24 hpf (TC_50_: 10 *μ*g/mL) and 48 hpf (TC_50_: 20 *μ*g/mL). Yolk-sac oedema and spine and the average embryonic heart rate was also decreased in embryos exposed to 25, 50, or 100 *μ*g/mL	[31]	Not compared
15	Radix *Sophorae tonkinensis*	Shandougen	Dried root fractions	Cure infectious and inflammatory diseases	RSTE and RST active fractions in zebrafish, concentration-dependent mortality were demonstrated (LC_50_ has not calculated)	Pericardial oedema and/or reduced heart rates were observable in different fractions of RSTE and RST	[32]	Compared with mice and similar results obtained
16	*Euphorbia kansui*		Dried root of Euphorbia kansui (KS-1) and Euphorbia kansui fry-baked with vinegar (KS-2)	Cancer, pancreatitis and intestinal obstruction	The LC_50_ value for *Euphorbia kansui* (KS-1) and fry-baked with vinegar KS-2 was reported as 2.78 ± 0.86 *μ*g/mL and 6.62 ± 1.24 *μ*g/mL, respectively	Pericardial oedema and scoliosis	[33]	Not compared

**Table 3:** Evaluation of the toxicity of phytochemical constituents of herbal medicine using the Zebrafish embryotoxicity model.

No.	Name of constituent	Phytochemical group	Herbal plant and part	Medicinal value	Toxic effects		Reference	Toxicity compared with other assays
					Survival/mortality rate	Teratogenic and other toxic effects		
1		Five anthraquinones, seven anthrones, and two naphthols	*Polygonum multiflorum Thunb*., whole plant	Antiageing, antihyperlipidaemia, antioxidant, anti-inflammatory, anticancer, hepatoprotective, and immunomodulating effects	LD50 values have been calculated for each compound at 48, 72 and 96 hpf	Notochord malformations were observed	[34]	Not compared
2	Matrine	Alkaloids	Kushen in traditional Chinese medicine root of Sophora flavescens	Possessing a variety of pharmacological effects such as anti-inflammation, antivirus, antitumour, and antiarrhythmic activities	EC_50_ and LC_50_ values at 145 and 240 mg/L	Oedema, growth retardation has been observed after 48 hrs concentrations below those causing lethality and malformations, indicating a neurotoxic potential of both drugs	[35]	Not compared
3	Sophocarpine.				EC_50_ and LC_50_ values 87.1 and 166 mg/L			
4	Celastrol	Terpenoid	Thunder God vine *Tripterygium wilfordii* Hook F	Antioxidant and anti-inflammatory activities, neurodegenerative diseases and anticancer	Dose-dependent and the LC_50_ values of celastrol on embryos were approximately 1.40 *μ*M	Several developmental abnormalities, including no blood flow, oedema in pericardial sac, and tail malformation were reported in embryos EC50 for tail malformation was 0.66 *μ*M at 72 hpf	[36]	Not compared
5	Emodin	An anthraquinone derivative	Rhubarb root and bark of many plants of the genus *Rhamnus*.	Antidiabetic, antinociception, anticancer and cholesterol reduction potential	Dose-related increase in mortality, with significant death of embryos at 0.25 *μ*g/mL. The LD_50_ value (at 72 hpf) −0.20 *μ*g/mL	Oedema, crooked trunk, and abnormal morphogenesis of some organs, such as statolith, swimming bladder, and yolk syncytium were reported in embryo treated with 0.1–1.5 *μ*g/mL emodin	[37]	Not compared
6	Cannabidiol (CBD)	Cannabis	*Cannabis sativa* whole plant	Neuropsychiatric disorders	CBD all concentrations did not show significant morphological demormaties. 300 *μ*g/L have significantly delayed the hatching of the embryo	Embryos exposed to CBD 20–300 *μ*g/L were 1.4 up to 1.7-fold more active when compared with the control. But difference in acetylcholinesterase	[38]	Not compared
7	Aristolochic acid (AA)		*Aristolochia* or *Asarum*.	Arthritis, gout, and festering wounds	No significant difference in survival rate between test and controls	AA-treated (10 ppm) embryos significantly reduced glomerular filtration rates compared with the control. Malformed kidney phenotypes, curved, cystic pronephric tubes, pronephric ducts, and cases of atrophic glomeruli were reported	[39]	Not compared
8	Psoralen		*Psoralea corylifolia* L	Psoriasis, vitiligo, osteoporosis, osteosarcoma, bone fracture, and osteomalacia	The values of LC_50_, LC_10_, and LC_1_ at 96 hpf were determined to be 18.24, 13.54, and 10.61 μM. The hatching rate in the 13.54 mM psoralen group (70%)	Yolk retention, swim-bladder deficiency, pericardial oedema, and curved body shape were observed at 24 to 96 hpf in psoralen-treatment embryos.	[40]	Not compared
9	Isofraxidin 7-O-(6'-*O-p*-coumaroyl)-*β*-glucopyranoside		*Artemisia capillaris* Thunberg	Enhanced pigmentation.	Greater than 90% of the treated embryos survived, which did not differ significantly from the control group.	The results revealed compound 1 (25 μM) treated embryos had no developmental defects and displayed normal cardiac function, indicating that this compound enhanced pigmentation without producing toxicity	[41]	Not compared
10	Evodiamine	Bioactive alkaloid	*Evodia rutaecarpa*	Abdominal pain, headache, menstrual problems, vomiting, and diarrhea	Concentrations ≥400 ng/mL significantly increased the lethality reached 100% at 1600 ng/mL evodiamine	10% lethal concentration of 354 ng/mL and induced cardiac malfunction, as evidenced by changes in heart rate and circulation, and pericardial malformations	[42]	Primary cultured neonatal rat cardiomyocytes
11	Tanshinone IIA (Tan-IIA)	Diterpene quinone	*Salvia miltiorrhiza Bunge*	Recommended for cardiovascular disease exhibits various pharmacological activities, including anti-inflammatory, antioxidative, antifibrosis, modulation of collagen metabolism, and antitumour	The LC_50_ values in the dechorionated embryo group at 72 hpf and 96 hpf were 18.5 *μ*M and 12.8 *μ*M, respectively. Normal embryos were less sensitive	Pericardial oedema at 6 *μ*M for 96 hpf and spinal curvature higher concentrations were cardiotoxic	[43]	Not compared
12	Gambogic acid (GA)		*Garcinia hanburyi Hook*.f.,	Anticancer	The LC_50_, LC_10_, MNLC and EC_50_ values were calculated as 1.76 *μ*M, 0.8 *μ*M, 0.5 *μ*M and 0.723 *μ*M, respectively	GA at 0.5–1.0 *μ*M caused specific fin developmental defect with the phenotype resembled those caused by thalidomide	[44]	Not compared
13	Aconitine (AC), Mesaconitine (MAC,) Hypaconitine (HAC) 14-A-benzoylaconine (BAC), 14-a-Benzoylmesaconine (BMAC) Benzoylhypaconine (BHAC)	Diterpene alkaloids (Das) Monoester diterpene alkaloids (MDA)	*Aconitum, Delphinium, Consolida*, and *Spiraea* species	Anti-inflammation, antidepressant, antiarrhythmia, antiplatelet aggregation, and antimalarial properties	Not investigated	Diterpenes including AC, MAC, and HAC exhibited serious organic and functional toxicities in zebrafish embryos compared with that of monoester diterpene alkaloid	[45]	Not compared
